# Correlation between variants of the *CREB1* and *GRM7* genes and risk of depression

**DOI:** 10.1186/s12888-022-04458-1

**Published:** 2023-01-03

**Authors:** Li Wang, Xingming Tang, Peng Liang, Chuan Zhou, Yingjie Sun, Yundan Liang

**Affiliations:** 1 Department of Anesthesiology, the First Affiliated Hospital of Chengdu Medical College, Chengdu, Sichuan China; 2 Chengdu Medical College, Chengdu, Sichuan China; 3 Department of Pathology and Pathophysiology, Faculty of Medicine, Chengdu Medical College, Chengdu, Sichuan China

**Keywords:** Polymorphism, Depression, Glutamate receptor 7, *cAMP*-response element-binding protein 1

## Abstract

The pathogenesis of depression involves *cAMP*-response element binding protein1 (*CREB1*) and metabotropic glutamate receptor 7 (*GRM7*), and their genetic polymorphisms may affect susceptibility to depression. The purpose of this study was to investigate whether the *CREB1 *polymorphisms rs2253206 and rs10932201 and the *GRM7* polymorphism rs162209 are associated with the risk of depression. Using polymerase chain reaction-restriction fragment length polymorphism and DNA sequencing, we analyzed the *rs2253206*, rs10932201, and rs162209 frequencies in 479 patients with depression and 329 normal controls. The results showed that the rs2253206 and rs10932201 polymorphisms were significantly associated with an increased risk of depression. However, no association was found between rs162209 and depression risk. When the data were stratified for several disease-related variables, none of the three polymorphisms were found to be correlated to onset, disease severity, family history, or suicidal tendency. Thus, the present findings indicate that the *CREB1* polymorphisms rs2253206 and rs10932201 may be related to the occurrence of depression.

**Supplementary Information:**

The online version contains supplementary material available at 10.1186/s12888-022-04458-1.

## Introduction

Depression is a common psychiatric disorder characterized by widespread and persistent depression and loss of interest [1]. Previous studies have shown that depression has become the second most common disease after cardiovascular disease [2, 3]. Depression is not only a burden to individuals, but is also a burden to society because it is associated with a 20 times higher suicide rate than that in normal individuals [4, 5]. Previously, a large number of studies have clarified that environmental, genetic, endocrine, and other factors together lead to the occurrence of depression [2], but the specific cause of depression is not clear. Therefore, the pathogenesis of depression still needs further study.

The role of genetics in increasing susceptibility to depression is recognized [6]. Cyclic adenosine monophosphate response element binding protein (*CREB1*)—a transcription factor that controls the transcription of numerous neuronal expressed genes—has been shown to be related to both the pathogenesis and treatment of depression [7, 8]. The *CREB1* gene is located on chromosome 2q34 and encodes a protein that is a member of the leucine zipper family of DNA-binding proteins [9]. Daniela et al. reported that *CREB1* plays an antidepressant role by regulating the expression of certain genes [10]. Additionally, studies have shown that *CREB1* plays a role in the effects of antipsychotics and mood stabilizers [11–13]. Several standard antidepressant treatment agents, including norepinephrine reuptake inhibitors and selective serotonin reuptake inhibitors, were found to result in elevated *CREB1* activity in the hippocampus [9, 14, 15]. With regard to depression, Serretti et al. investigated five single nucleotide polymorphisms (SNP*s*) in *CREB1* in a sample of depression patients for their association with antidepressant response, remission, and treatment resistance, and they found that some genetic polymorphisms in *CREB1* could be related to treatment resistance [16].

Apart from *CREB1*, metabotropic glutamate receptor 7 (*GRM7*), which mediates the effect of glutamate on neurotransmitter release and cell excitability [17, 18], has been found to be related to depression. *GRM7* is located at 3p26 and spans over 900 KB, and is expressed in many regions of the human central nervous system [19]. *GRM7* plays a protective role against neuronal excitotoxicity by inhibiting the secondary messenger adenylate cyclase and reducing the activity of the N-methyl-d-aspartate receptor. It can also regulate the release of l-glutamate and *GABA*, affect mood, and lead to anxiety and even depression [20, 21]. Wierońska et al. found that the *GRM7* agonist AMN082 had an antidepressant effect on mice that could be blocked by *GRM7* gene knockout [22]. They also found that chronic antidepressant treatment of rodents with citalopram reduced *GRM7* immunoreactivity in the hippocampus and frontal cortex [22]. Further, Zhou’s study showed that *GRM7* was involved in the regulation of antidepressant response [23]. The amygdala and hippocampus in the brain are known to play a key role in alleviating anxiety and antidepressant, and *GRM7* is abundant in these regions [24]. Thus, *GRM7* may be involved in the regulatory circuit that affects anxiety and/or depressive behavior [24]. With regard to depression, a meta-analysis showed that the rs162209 polymorphism in the *GRM7* gene was closely associated with depression [25].

Previous work has shown that the rs2253206 and rs10932201 in *CREB1* and rs162209 in *GRM7* may light on the pathogenesis of depression [7, 26, 27]. However, there are few reports on the genetic susceptibility of *CREB1* SNPs rs2253206 and rs10932201 and *GRM7* SNPs rs162209 involved in depression. In order to verify their correlation with depression and explore their possible mechanisms, we investigated the association of the *CREB1* SNP*s* rs2253206 and rs10932201 and the *GRM7* rs162209 with first onset, family history, and suicidal tendency in patients with depression.

## Materials and methods

### Study participants

This case-control study included 480 patients with depression and 329 healthy controls who were recruited from Jining Psychiatric Hospital and the Sichuan Provincial People's Hospital between March 2018 and December 2019. Chengdu Medical College ethics committee approved of the study (NO.201815), and all the participants signed a complete written consent form after they were informed of the purpose of the project. The patients were diagnosed based on the DSM-IV criteria, and the ratings for symptom severity were evaluated using the 24-item version of the Hamilton rating scale for depression (HAMD-24). The structured interview of depression includes information about mood, insomnia, interests, general condition, and suicide attempt. As described in the previous study of Liang et al., the exclusion criteria were neurodegenerative diseases (for instance, Alzheimer's disease and Parkinson's disease), cognitive impairment, other mental disorders (such as drug abuse), neurological diseases, infections (acute or chronic), thyroid dysfunction, pregnancy, and lactation [28]. The following information was obtained from the participants’ medical records: age, gender, age of onset, HAMD score, pulse rate, depressive episode, family history, suicide attempt, and whether it was their first episode (yes or no). The control group included healthy volunteers without psychiatric conditions who consulted the hospital for physical examination during the same period, and the same exclusion criteria were applied. The control participants were frequency-matched with the patients by age, gender, ethnicity, and living area. The mean age of the depression patients (139 males and 341 females) was 41.8 ± 17.9 years, while the mean age of the controls (105 males and 224 females) was 44.0 ± 16.9 years (Table 1).

**Table 1:** Characteristics of the study population

Variables	Controls (*n* = 329)	Patients (*n* = 480)	*P* value
Age (years)	44.0 ± 16.9	41.8 ± 17.9	0.08
Gender, n (%)			
Male	105 (31.9)	139 (29.0)	0.37
Female	224 (68.1)	341 (71.0)	
Age of onset (years)		36.8 ± 16.9	
Pulse rate		80.6 ± 11.4	
Depressive episode (%)			
Severe		252 (52.5)	
Mild/moderate		228 (47.5)	
Family history (%)			
Positive		94 (19.6)	
Negative		386 (80.4)	
Suicide attempt			
Yes		292 (60.8)	
No		188 (39.2)	
First episode (%)			
Yes		248 (51.7)	
No		232 (48.3)	

### Genotyping

**Fig. 1:**
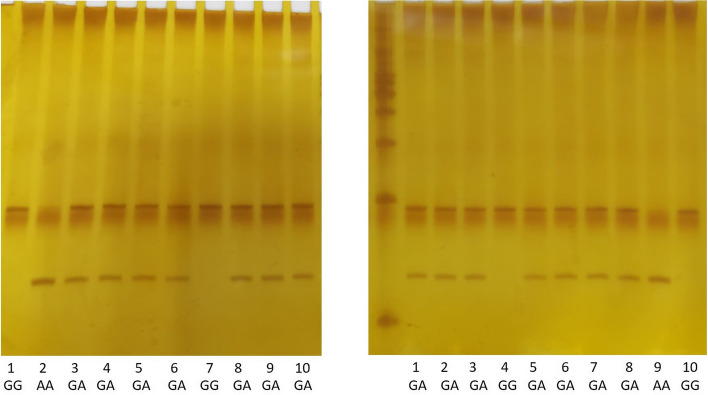
Results of enzymatic digestion of rs2253206 PCR products

**Fig. 2:**
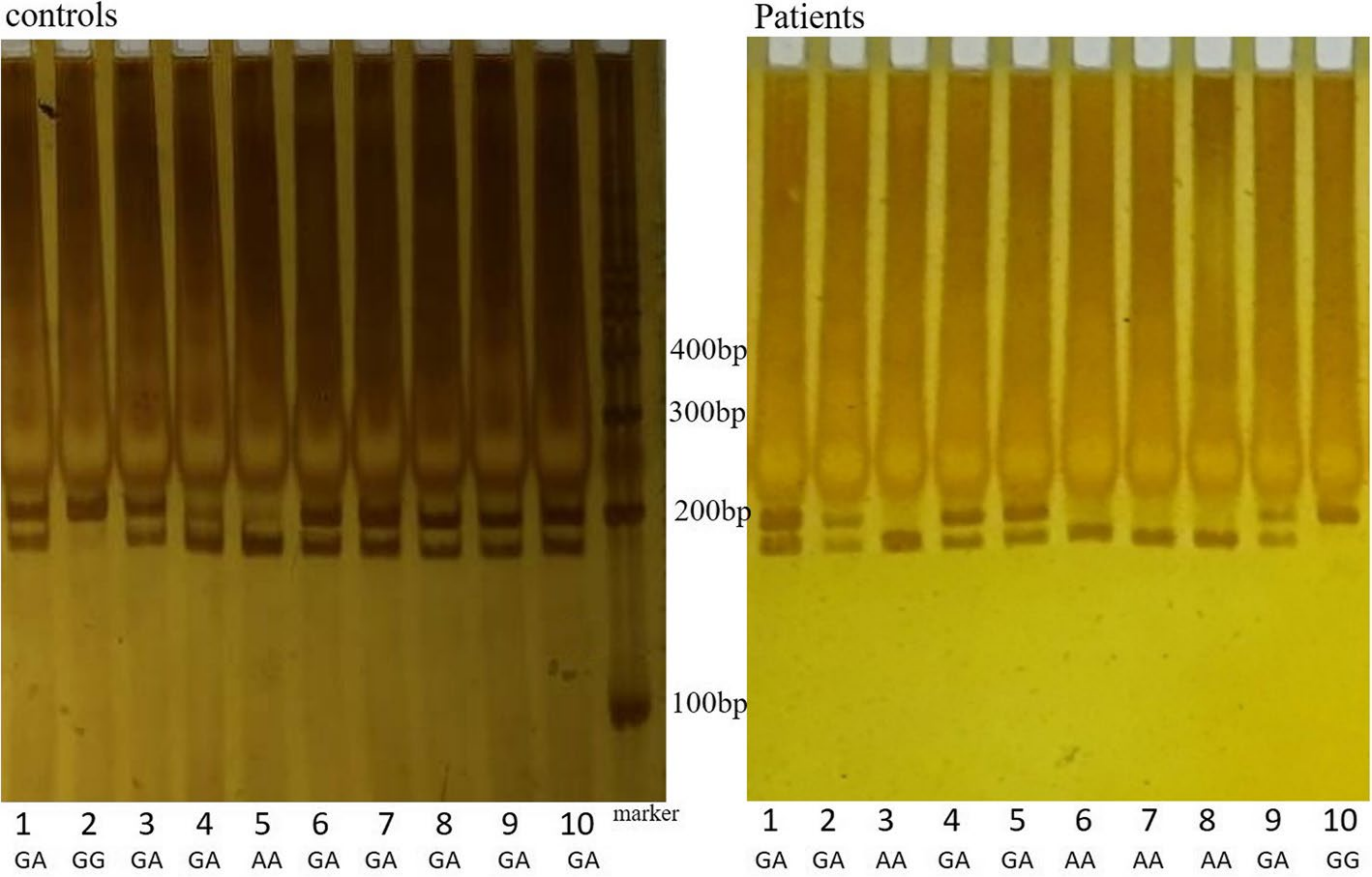
Results of enzymatic digestion of rs10932201 PCR products

Peripheral blood samples were collected in EDTA-containing test tubes, and genomic DNA was extracted using a DNA isolation kit according to the manufacturer’s instructions (Bioteke, Beijing, China). Information about candidate genes and SNPs involved in this study are shown in Table 2. The rs2253206 and rs10932201 SNP*s* were genotyped by polymerase chain reaction-restriction fragment length polymorphism (PCR-RFLP) analysis. The primers for rs2253206 and rs10932201 are shown in Table 2. PCR was performed under the following conditions: 98°C for 5 min, followed by 35 cycles of 98°C for 30 s, annealing for 30 s, 72°C for 10 s, and 72°C for 10 min. The annealing temperature for rs2253206 was 58°C, while that for rs10932201 was 57°C. The PCR product of rs2253206 was digested for 4 h at 37°C with Msel (New England Biolabs, Ipswich, MA, USA), and the rs10932201 product was digested with *Hinf*l (Thermo Fisher Scientific, Waltham, USA) under the same conditions. After digestion, for rs2253206, the heterozygosis GA genotype was indicated by bands at 191, 128 and 63 bp; the GG genotype was indicated by a band at 191 bp; and the AA genotype was indicated by bands at 128 and 63 bp (Fig. 1). For rs10932201, the heterozygosis GA genotype was indicated by bands at 196, 176 and 20 bp; the GG genotype was indicated by a band at 196 bp; and the AA genotype was indicated by bands at 176 and 20 bp (Fig. 2). DNA sequencing was used to confirm the genotyping results. The rs162209 polymorphism was analyzed by DNA direct sequencing.

**Table 2:** Information about candidate genes and SNPs involved in this study

Gene	*CREB1*	*CREB1*	*GRM7*
SNP ID	rs2253206	rs10932201	rs162209
Primer sequence (forward)	5′-GTGCTGTTGCTAGGGAGAGG-3′	5′-GTGATCCCGGGTAAACACAG-3′	5′-GGAGGCAGGTTTCTGACTTG-3′
Primer sequence (reverse)	5′-GGCATTTACACATGCCCTTC-3′	5′-CAACCAGGATGGTGAAGAGG-3′	5′-AACGTCCCAGGATGTGATCT-3′
Genotype techniques	PCR–RFLP	PCR–RFLP	Next-generation sequencing
Length of PCR products(bp)	191	196	224
Restriction endonuclease	Msel	Hinfl	-
Length of digested product (bp)	128 and 63	176 and 20	-

*SNP* single nucleotide polymorphisms, *PCR–RFLP* polymerase chain reaction-restriction fragment length polymorphism

### Statistical analyses

The SPSS26.0 statistical software (SPSS Inc., Chicago, Illinois, USA) was used for data analysis. The rs2253206, rs10932201, and rs162209 genotype frequencies were determined by direct counting. Concordance with the Hardy–Weinberg equilibrium was assessed with the χ2 test. The rs2253206, rs10932201, and rs162209 genotype frequencies in the cases and controls were examined using the χ^2^ test, and the correlation between the three polymorphisms and depression risk was assessed using odds ratios (ORs) and 95% confidence intervals (CIs). The comparison was analyzed using different inheritance patterns, such as codominant, dominant, and recessive genetic model. A *P* value of <0.05 was considered to indicate statistical significance.

## Results

The genotype frequencies of the three polymorphisms in the control participants and patients with depression are presented in Table 3. Due to poor DNA quality in some cases, individual samples could not be genotyped successfully and were thus excluded from the analysis. The genotype distribution in the control group did not deviate from the Hardy–Weinberg equilibrium (*P* ≥ 0.05). In the codominant model, the rs2253206 AA genotype showed significantly different distribution between the depression patients and controls (adjusted OR = 2.00, 95% CI = 1.15–3.49, *P* value = 0.01). Similarly, in the recessive model, the AA genotype also showed significantly different frequency between depression patients and controls (adjusted OR = 1.93, 95% CI = 1.13–3.29, *P* = 0.01). Accordingly, with regard to allele distribution, too, the frequency of A was significantly higher in the depression group (adjusted OR = 1.25, 95% CI = 1.00–1.56, *P* = 0.05). With regard to the rs10932201 polymorphism, the GA genotype in the codominant model and the GA/GG genotype in the dominant model showed significantly higher frequencies in the depression group (GA: adjusted OR = 1.81, 95% CI = 1.27–2.58, *P* = 0.001; GA/GG: adjusted OR = 0.74, 95% CI = 0.52–1.04, *P* = 0.002), as did the G allele (adjusted OR = 1.29, 95% CI = 1.01–1.65, *P* = 0.04). In contrast, there was no obvious difference in the distribution of the rs162209 genotypes between depression patients and controls.

**Table 3:** Association of the rs2253206, rs10932201, and rs162209 polymorphisms with depression risk

Models	Polymorphisms	Controls, n (%)	Patients, n (%)	Adjusted OR (95% CI)	Adjusted *P* value
	rs2253206	*n* = 300	*n* = 479		
Codominant	GG	140 (46.7%)	201 (42.0%)	1.00 (Ref)	
	GA	140 (46.7%)	221 (46.1%)	1.08 (0.79–1.46)	0.64
	AA	20 (6.7%)	57 (11.9%)	2.00 (1.15–3.49)	0.01
Dominant	G/G	140 (46.7%)	201 (42.0%)	1.00 (Ref)	
	GA/AA	160 (53.3%)	278 (58.0%)	1.20 (0.89–1.60)	0.23
Recessive	GG/GA	280 (93.3%)	422 (88.1%)	1.00 (Ref)	
	AA	20 (6.7%)	57 (11.9%)	1.93 (1.13–3.29)	0.01
Allele	G	420 (70.0%)	623 (65.0%)	1.00 (Ref)	
	A	180 (30.0%)	335 (35.0%)	1.25 (1.00–1.56)	0.05
	rs10932201	*n* = 301	*n* = 480		
Codominant	AA	158 (52.5%)	178 (37.1%)	1.00 (Ref)	
	GA	112 (37.2%)	252 (52.5%)	1.81 (1.27–2.58)	0.001
	GG	31 (10.3%)	50 (10.4%)	1.02 (0.55–1.90)	0.94
Dominant	AA	158 (52.5%)	178 (37.1%)	1.00 (Ref)	
	GA/GG	143 (47.5%)	302 (62.9%)	0.74 (0.52–1.04)	0.002
Recessive	AA/GA	270 (89.7%)	430 (89.6%)	1.00 (Ref)	
	GG	31 (10.3%)	50 (10.4%)	0.87 (0.50–1.51)	0.63
Allele	A	428 (71.1%)	608 (63.3%)	1.00 (Ref)	
	G	174 (28.9%)	352 (36.7%)	1.29 (1.01–1.65)	0.04
	*rs162209*	*n* = 329	*n* = 480		
Codominant	AA	206 (62.6%)	321 (66.9%)	1.00 (Ref)	
	AG	109 (33.1%)	136 (28.3%)	0.72 (0.50–1.03)	0.07
	GG	14 (4.3%)	23 (4.8%)	0.86 (0.38–1.98)	0.73
Dominant	AA	206 (62.6%)	321 (66.9%)	1.00 (Ref)	0.08
	GA/GG	123 (37.4%)	159 (33.1%)	0.74 (0.52–1.04)	
Recessive	AA/GA	315 (95.7%)	457 (95.2%)	1.00 (Ref)	0.99
	GG	14 (4.3%)	23 (4.8%)	1.01 (0.45–2.23)	
Allele	A	521 (79.2%)	778 (81.2%)	1.00 (Ref)	
	G	137 (20.8%)	182 (19.0%)	0.81 (0.61–1.07)	0.15

OR and *P* value were adjusted by age and gender

Stratification analyses according to depressive episodes (severe vs. mild/moderate), suicide attempt (yes vs. no) and first episode (yes vs. no) showed that the rs10932201, *rs2253206*, and rs162209 polymorphisms were not significantly correlated with these variables (*P* > 0.05) (Table 4).

**Table 4:** Stratified analyses of the rs10932201, rs2253206, and rs162209 polymorphisms in depression patients

Variables	Frequency		Adjusted OR (95% CI)	Adjusted *P* value
	%	%		
rs10932201 Depressive episode	Severe	Mild		
AA	102 (40.5)	76 (33.3)	1.00 (Ref)	
GA	124 (49.2)	128 (56.1)	1.38 (0.93–2.05)	0.10
GG	26 (10.3)	24 (10.5)	1.32 (0.69–2.53)	0.40
GA/GG	150 (59.5)	152 (66.7)	1.37 (0.94–2.00)	0.10
Suicide attempt	Yes	No		
AA	115 (39.4)	63 (33.5)	1.00 (Ref)	
GA	142 (48.6)	110 (58.5)	1.44 (0.95–2.18)	0.09
GG	35 (12)	15 (8)	0.81 (0.39–1.68)	0.56
GA/GG	177 (60.6)	125 (66.5)	1.32 (0.88–1.97)	0.18
First episode	Yes	No		
AA	97 (39.1)	81 (34.9)	1.00 (Ref)	
GA	127 (51.2)	125 (53.9)	1.17 (0.79–1.73)	0.43
GG	24 (9.7)	26 (11.2)	1.45 (0.75–2.81)	0.27
GA/GG	151 (60.9)	151 (65.1)	1.20 (0.82–1.76)	0.34
rs2253206				
Depressive episode	Severe	Mild		
GG	107 (42.5)	94 (41.4)	1.00 (Ref)	
GA	118 (46.8)	103 (45.4)	1.01 (0.68–1.50)	0.96
AA	27 (10.7)	30 (13.2)	1.24 (0.68–2.26)	0.49
GA/AA	145 (57.5)	133 (58.6)	1.05 (0.73–1.53)	0.78
Suicide attempt	Yes	No		
GG	120 (41.2)	81 (43.1)	1.00 (Ref)	
GA	140 (48.1)	81 (43.1)	0.87 (0.57–1.32)	0.51
AA	31 (10.7)	26 (13.8)	1.26 (0.67–2.38)	0.48
GA/AA	171 (58.8)	107 (56.9)	0.93 (0.63–1.38)	0.73
First episode	Yes	No		
GG	107 (43.1)	94 (40.7)	1.00 (Ref)	
GA	112 (45.2)	109 (47.2)	1.17 (0.79–1.74)	0.43
AA	29 (11.7)	28 (12.1)	1.06 (0.58–1.93)	0.86
GA/AA	140 (56.9)	136 (59.3)	1.14 (0.79–1.66)	0.48
rs162209				
Depressive episode	Severe	Mild		
AA	163 (64.7)	158 (69.3)	1.00 (Ref)	
GA/GG	89 (35.3)	70 (30.7)	0.83 (0.56–1.22)	0.34
Suicide attempt	Yes	No		
AA	191 (65.4)	130 (69.2)	1.00 (Ref)	
GA/GG	101 (34.6)	58 (30.9)	0.87 (0.58–1.32)	0.52
First episode	Yes	No		
AA	162 (65.3)	159 (68.5)	1.00 (Ref)	
GA/GG	86 (34.7)	73 (31.5)	0.90 (0.61–1.32)	0.58

OR and *P* value were adjusted by age and gender

## Discussion

In this study, we explored the association between SNPs of candidate genes *CREB1* and *GRM7* in depression among Chinese people. We found that the *CREB1* rs2253206 AA and rs10932201 GA genotypes were associated with an increased risk of depression. Specifically, the rs2253206 AA genotype in the recessive model showed a significantly different frequency between depression patients and controls, indicating that the absence of rs2253206 G allele has a significant correlation with MDD risk compared with the G allele. Similarly, the rs10932201 GA/GG genotype in the dominant model showed a significantly different frequency between depression patients and controls, indicating that the rs10932201 G allele has an obvious correlation with MDD risk. These findings indicate that the *CREB1* rs2253206 and rs10932201 polymorphisms may be associated with the occurrence of depression in the Chinese population.

As a transcription factor, *CREB1* participates in synaptic and neuronal plasticity on the basis of *BDNF* pathway [9]. *CREB-BDNF* pathway is closely related to many neurobiological processes, including synapse and neural plasticity, which may be a potential mechanism for the occurrence and development of depression [8]. Our present findings are consistent with the findings of previous studies, and verifying the correlation between *CREB1* and depression in Chinese people [7, 27].

Previous studies on rs2253206 were mostly about panic disorder and memory, but less about depression [8, 29, 30]. Interestingly, in Ma et al.'s study, GG genotype of rs2253206 was susceptible to depression when exposed to high negative life events, but our study found that rs2253206 AA genotype increases the susceptibility to depression [7]. However, there is no research report on the susceptibility of rs10932201 to depression. This may be related to the different characteristics of the selected population, such as age, gender, or our limited sample size. In the future, we will verify our findings in a larger sample size and different populations. Additionally, a meta-analysis showed that an SNP located in *CREB1* was associated with depression, and that a decrease in *CREB1* expression may be a risk factor for depression [9]. It has been found that antidepressants may reduce the level of *CREB1* protein by affecting hippocampal function and activity [31, 32]. On the other hand, gene modification to increase the level of *CREB1* protein in the mouse hippocampus was also found to produce an antidepressant effect [33]. These contradictory results imply that further study of *CREB1* is urgently needed to describe the overall picture of the genetic and biological basis of *CREB1* and its protein product in susceptibility to depression. Therefore, it will be our next research goal.

The present findings showed that the *GRM7* SNP rs162209 did not increase susceptibility to depression. However, other studies have reported that *GRM7* is associated with depression [17, 34], and Genome-wide association study (GWAS) and meta-analyses have also shown that *GRM7* is associated with depression [35–37]. Further, Jun et al. proposed the hypothesis that *GRM7* affects mood by regulating glutamate as a supplement to the monoamine hypothesis of depression [38]. Specifically, change in the glutamine/glutamate ratio is associated with the onset of depression [39]. In 2010, a study reported that the SNP rs162209 in the *GRM7* gene was related to depression, but there have been no subsequent reports on the role of this SNP in depression [40]. In the current study, we failed to find any association of the SNP of rs162209 with the susceptibility to depression. Few studies on the relationship between SNPs of *GRM7* and depression were reported, and thus comparison analysis between our data and others cannot be performed and the negative conclusion should be made with caution. Therefore, irrespective of the present findings, other SNPs of *GRM7* should be examined to explore the potential relationship between *GRM7* and depression.

In the present study, the polymorphisms examined were not correlated with disease severity, onset, family history, or suicidal tendency.

One of the main limitations of this study is that we have only focused on a small number of SNPs in a limited sample, and this may have led to a false null hypothesis. Our research has not found that rs10932201, rs2253206 and rs162209 polymorphisms are significantly related to the variables of depressive episodes, suicide attempt and first episode, which may be caused by small sample size. In addition, the control group we selected may not represent the general population, even though there is no evidence of deviation from the Hardy-Weinberg equilibrium in this group.

In order to reveal the biological basis of the SNPs in the occurrence and development of depression, future research could target these SNPs and examine in bigger cohorts and different populations, and explore the expression of related genes and the impact of protein products on depression.

This study provides preliminary evidence for the correlation between rs2253206 and rs10932201 polymorphisms of *CREB1* and susceptibility to depression. In the future, these SNPs should be examined in bigger cohorts in order to understand how they affect depression susceptibility. However, there was no evidence of the *GRM7* polymorphism rs162209 and its effect on susceptibility to depression.

## Supplementary Information

**Additional file 1: Supplementary Table 1.** The results of the genotyping call rates and Hardy-Weinberg equilibrium tests of the rs2253206, rs10932201, and rs162209 polymorphisms.

## Data Availability

All data generated or analyzed during this study are included in this published article.
