# Supplementary material for: Correlation between variants of the CREB1 and GRM7 genes and risk of depression
Source: BMC Psychiatry. 2023 Jan 3;23:3. doi: 10.1186/s12888-022-04458-1 (PMC9811780; doi:10.1186/s12888-022-04458-1)
Supplement: Supplementary file 1 — Additional file 1: Supplementary Table 1. The results of the genotyping call rates and Hardy-Weinberg equilibrium tests of the rs2253206, rs10932201, and rs162209 polymorphisms. [file 12888_2022_4458_MOESM1_ESM.docx]

Supplementary Table 1. The results of the genotyping call rates and Hardy-Weinberg equilibrium tests of the rs2253206, rs10932201, and rs162209 polymorphisms.

|  | Polymorphism | Controls, n (%) | Patients, n (%) |
| --- | --- | --- | --- |
|  | rs2253206 |  |  |
| Genotyping | GG | 140 (46.7%) | 201 (42.0%) |
|  | GA | 140 (46.7%) | 221 (46.1%) |
|  | AA | 20 (6.7%) | 57 (11.9%) |
| Hardy-Weinberg equilibrium(P value) |  | 0.05 | 0.75 |
| Genotyping call rate |  | 91.19% | 99.80% |
|  |  |  |  |
|  | rs10932201 |  |  |
| Genotyping | GG | 31 (10.3%) | 50 (10.4%) |
|  | GA | 112 (37.2%) | 252 (52.5%) |
|  | AA | 158 (52.5%) | 178 (37.1%) |
| Hardy-Weinberg equilibrium(P value) |  | 0.10 | 0.004 |
| Genotyping call rates |  | 91.49% | 100% |
|  |  |  |  |
|  | rs162209 |  |  |
| Genotyping | GG | 14 (4.3%) | 23 (4.8%) |
|  | GA | 109 (33.1%) | 136 (28.3%) |
|  | AA | 206 (62.6%) | 321 (66.9%) |
| Hardy-Weinberg equilibrium(P value) |  | 0. 93 | 0.09 |
| Genotyping call rate |  | 100% | 100% |
